# Editorial

**Published:** 1963-01

**Authors:** 


### EDITORIAL

The Journal has reverted to its original title, under which it made its name, and which most truly describes its character. From its beginning in 1883 it bore the subtitle "A Journal of the Medical Sciences for the West of England and South Wales", and justified this extension of its field by publishing some contributions and items of medical news from a wide area of the West Country. In 1952 it was hoped to establish more firmly this aspect of the Journal's work by changing its title to "The Medical Journal of the South West".

The change in name in 1952 came soon after the formation of the "South-West Region" as a division of the country under the newly established National Health Service, and there was a feeling that the new title would bring the Journal into Line as a regional publication, not committed to Bristol alone. We must honour the idealism of our predecessors in taking this decision. But experience over the last ten years has shown that while the Region may have functioned well as an administrative unit, there was not the demand for a regional medical journal that had been expected. In fact there has been little or no change in the character, contents, or circulation of the Journal, and it has continued to receive its chief support and material from members of the Society in Bristol, though there have been notable and welcome contributions from elsewhere in the south-west, as there always had been. It would seem fitting therefore that the Journal should resume its former title which, outside Britain, has the merit of indicating clearly its place of origin. This change has been agreed to by the Society which gave birth to the Journal.

In medical journalism, the trend at present is away from local publications. A local journal must justify its existence on grounds that are different from those justifying the existence of specialized or general national medical journals. There are several reasons for thinking that the Bristol Medico-chirurgical Journal still has a duty to perform.

Firstly, it has a long tradition. Continuous publication for 80 years is no mean feat, and has created an historical document of some value; to interrupt this, except for some weighty reason, would be unfair to our successors. Secondly, sufficient addresses and communications are made to local societies to enable the Editors to select those that would be welcome in print both for contemporary reading and as a record of what is said and done locally. Thirdly, the Journal has a duty to accept the early writings of our junior colleagues, and to help in the preparation of papers by those who one day may outshine us all. History, contemporary interest, and education—these are our objectives.

The present number happily exemplifies this. The Presidential Address embodies the tradition of a learned and humanitarian Society. The Long Fox Lecture contains the work of a distinguished graduate of Bristol University. A clinico-pathological conference and the reports of regional meetings of specialists record some of our daily activities; and finally a paper whose co-author is a medical student fulfills our last objective.

In presenting this newly-styled Journal to our members we remind them that all our objectives will be the more fully achieved if the needs of the Journal and its readers are borne in mind by those whose seniority and experience fits them to write for the instruction of others; by those who arrange and could report the activities of local medical groups; and by those to whom medical writing is as yet an untried venture. Its future success must depend upon the many, and not upon the few.

